# Unsupervised Analysis Based on DCE-MRI Radiomics Features Revealed Three Novel Breast Cancer Subtypes with Distinct Clinical Outcomes and Biological Characteristics

**DOI:** 10.3390/cancers14225507

**Published:** 2022-11-09

**Authors:** Wenlong Ming, Fuyu Li, Yanhui Zhu, Yunfei Bai, Wanjun Gu, Yun Liu, Xiaoan Liu, Xiao Sun, Hongde Liu

**Affiliations:** 1 State Key Laboratory of Bioelectronics, School of Biological Science and Medical Engineering, Southeast University, Nanjing 210096, China; 2 Division of Medical Image Computing, German Cancer Research Center (DKFZ), Im Neuenheimer Feld 280, 69120 Heidelberg, Germany; 3 Department of Breast Surgery, the First Affiliated Hospital of Nanjing Medical University, 300 Guangzhou Road, Nanjing 210029, China; 4 Collaborative Innovation Center of Jiangsu Province of Cancer Prevention and Treatment of Chinese Medicine, School of Artificial Intelligence and Information Technology, Nanjing University of Chinese Medicine, Nanjing 210023, China; 5 Department of Information, the First Affiliated Hospital of Nanjing Medical University, 300 Guangzhou Road, Nanjing 210029, China

**Keywords:** radiogenomics, radiomics, breast cancer, dynamic contrast-enhanced magnetic resonance imaging, tumor heterogeneity, prognosis

## Abstract

**Simple Summary:**

Dynamic contrast-enhanced magnetic resonance imaging (DCE-MRI) is an important approach for the diagnosis and evaluation of breast cancer (BC) in clinical practice. Recently, DCE-MRI-based radiomics studies have received widespread attention and application in BC research, such as in non-invasively predicting subtypes and recurrence risks. Therefore, we collected two radiogenomics cohorts of BC and identified and validated three novel imaging subtypes by unsupervised analysis in this work. In several external datasets, we found that breast tumors with larger sizes and showing rapid enhancement patterns generally had the worst prognostic outcomes. The bioinformatics analysis revealed significant differences in gene expression profiling and tumor microenvironment characteristics among the three imaging subtypes. These findings highlight the heterogeneity in BC imaging and its potential value as a clinical biomarker for BC and for achieving precision medicine in BC.

**Abstract:**

Background: This study aimed to reveal the heterogeneity of dynamic contrast-enhanced magnetic resonance imaging (DCE-MRI) of breast cancer (BC) and identify its prognosis values and molecular characteristics. Methods: Two radiogenomics cohorts (*n* = 246) were collected and tumor regions were segmented semi-automatically. A total of 174 radiomics features were extracted, and the imaging subtypes were identified and validated by unsupervised analysis. A gene-profile-based classifier was developed to predict the imaging subtypes. The prognostic differences and the biological and microenvironment characteristics of subtypes were uncovered by bioinformatics analysis. Results: Three imaging subtypes were identified and showed high reproducibility. The subtypes differed remarkably in tumor sizes and enhancement patterns, exhibiting significantly different disease-free survival (DFS) or overall survival (OS) in the discovery cohort (*p* = 0.024) and prognosis datasets (*p* ranged from <0.0001 to 0.0071). Large sizes and rapidly enhanced tumors usually had the worst outcomes. Associations were found between imaging subtypes and the established subtypes or clinical stages (*p* ranged from <0.001 to 0.011). Imaging subtypes were distinct in cell cycle and extracellular matrix (ECM)-receptor interaction pathways (false discovery rate, FDR < 0.25) and different in cellular fractions, such as cancer-associated fibroblasts (*p* < 0.05). Conclusions: The imaging subtypes had different clinical outcomes and biological characteristics, which may serve as potential biomarkers.

## 1. Introduction

Breast cancer (BC) has surpassed lung cancer as the most prevalent cancer in the world, placing a heavy burden on global healthcare each year [1]. The heterogeneity of BC has been noted in gene expression profiles, histology, and clinical outcomes for a long time, which have served as the basis of disease classification [2,3]. Based on the gene expression profile, BC can be defined as five PAM50 intrinsic molecular subtypes, including luminal-A, luminal-B, HER2-enriched, basal-like, and normal-like [4,5]. In the routine implementation, various histopathological receptors including estrogen receptor (ER), progesterone receptor (PR), human epidermal growth factor receptor 2 (HER2), and Ki67 are widely used to determine the clinical subtypes of BC, and guide the clinical decision-making [6,7]. These factors shape the distinct patterns of tumor proliferation, metastasis, and treatment resistance in patients.

Many studies have been performed to dissect the molecular and clinical heterogeneity of BC, but that alone is still not enough in the era of personalized medicine. Fortunately, the development of multi-omics offers the possibility to understand the whole spectrum of disease, especially using radiomics and radiogenomics, providing an emerging perspective to characterize a disease noninvasively [8,9]. Radiomics is now gaining increasing attention in cancer research, by integrating high-throughput quantitative medical imaging features into clinical decision support systems, such as malignancy diagnosis, clinical parameters prediction, and prognosis and treatment response prediction [10,11,12,13,14,15,16,17,18]. For example, a radiomics signature consisting of 24 computed tomography imaging features performed well in predicting lymph node metastasis in colorectal cancer [18], and recent work showed that magnetic resonance imaging (MRI) features were associated with the genomic subclones and could predict the clinical outcomes of BC [19]. In the clinic, various imaging techniques are applied to diagnose BC, such as ultrasound, mammography, and MRI; among these, dynamic contrast-enhanced magnetic resonance imaging (DCE-MRI) data are widely used in BC radiomics or radiogenomics studies for their advantages in high quality and three-dimension resolution. However, most studies focus on uncovering the relationship between medical imaging features and the molecular or clinical characteristics of the disease, and few have analyzed the disease heterogeneity in imaging phenotypes from an independent perspective [19,20,21,22,23,24,25].

**Figure 1:**
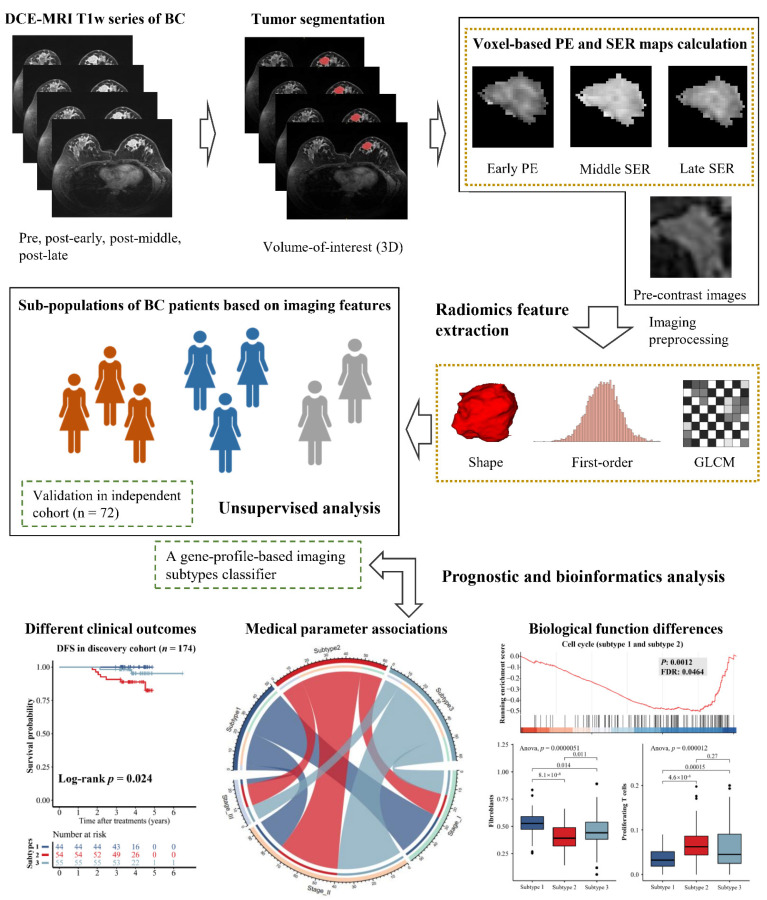
Workflow of this unsupervised analysis based on DCE-MRI radiomics features in BC patients. In this study, we took advantage of DCE-MRI techniques and calculated the voxel-based percentage enhancement (PE) and signal enhancement ratio (SER) maps for each patient. Two independent radiogenomics cohorts (*n* = 246) were collected to identify and validate the novel imaging subtypes. The clinical and biological associations of DCE-MRI subtypes were further investigated.

Although imaging features are important phenotypes, few studies have focused on disease heterogeneity in radiomics compared to genomics or transcriptomics. In this study, we hypothesized that imaging features could reflect BC heterogeneity. Therefore, we recruited two independent radiomics cohorts with both DCE-MRI and RNA-Seq data in this work, one as the imaging-subtype discovery cohort with 174 samples and the other as the imaging-subtype validation cohort with 72 samples. The representative imaging features from tumor regions were extracted to mine the potential imaging subtypes de novo. We further identified differences in clinical examination, imaging, and prognosis among the subtypes, and revealed the underlying reasons for this heterogeneity in terms of transcriptional activity and tumor immune microenvironment. The workflow is shown in Figure 1. Our findings demonstrated that the imaging subtypes with distinct clinical and molecular characteristics were reliable and reproducible, and were useful for the noninvasive prediction of outcome and biological functions of BC in the clinic.

## 2. Materials and Methods

### 2.1. Study Design

This study was designed as a multicentric exploratory study to investigate and validate the heterogeneity of quantitative DCE-MRI radiomics features in BC and to uncover the potential values in the prognosis of this kind of heterogeneity and the biological mechanisms behind it (Figure 1). To achieve this goal, an unsupervised analysis pipeline including the volume-of-interest (VOI) segmentation of tumor lesions, voxel-based percentage enhancement (PE) and signal enhancement ratio (SER) maps calculation, image normalization, resampling, radiomics feature extraction, and consensus clustering and bioinformatics analysis was conducted in two independent radiogenomics cohorts. The patients and technical details are reported below.

### 2.2. Radiogenomics Cohorts and Datasets

Two independent radiogenomics cohorts with both DCE-MRI and RNA-Seq data (as imaging-subtype discovery and validation cohorts, respectively) were recruited. Data from the imaging-subtype discovery cohort included preoperative T1-weighted DCE-MRI and RNA-Seq data collected between August 2016 and December 2018 in the local database. The imaging-subtype validation cohort is a public dataset collected from 4 medical centers, including the Memorial Sloan Kettering Cancer Center, the Mayo Clinic, the University of Pittsburgh Medical Center, and the Roswell Park Cancer Institute [26]. The DCE-MRI data were from the Cancer Imaging Archive (TCIA) and the RNA-seq data were part of a larger prognosis validation dataset, TCGA-BRCA (*n* = 1050), from the Cancer Genome Atlas (TCGA). The inclusion and exclusion criteria are displayed in Figure S1. Finally, the imaging-subtype discovery and validation cohorts included 174 and 72 patients, respectively. In addition, six external datasets of BC with only gene expression profiles (*n* = 1443) were downloaded from the Gene Expression Omnibus (GEO) database, including GSE1456, GSE3494, GSE7390, GSE20685, GSE25055, and GSE25065, which were used as prognosis validation datasets in this study.

### 2.3. Pathological Assessment

The clinical immunohistochemistry (IHC) results of ER, PR, HER2, Ki67 and IHC subtypes were determined for the patients in the discovery cohort. A patient with ER positive, HER2 negative, high PR expression (more than 20%), and low Ki67 expression (less than 20%) was regarded as luminal-A. Luminal-B patients were ER positive, HER2 negative, with low PR expression or high Ki-67 expression, or both ER and HER2 positive. ER, PR negative, and HER2 positive were HER2-positive, and, finally, patients with all negative IHC receptors were defined as triple negative BC (TNBC).

### 2.4. Imaging Parameters

The T1-weighted images were scanned in the axial position and acquired by Siemens TrioTim 3-Tesla scanner (Siemens Healthcare, Erlangen, Germany) in the imaging-subtype discovery cohort. The detailed parameters of most images were as follows: flip angle, 10 degrees; echo time, 15.7 ms; repetition time, 423 ms; field of view, 340 × 340 mm; slice thickness, 0.9 mm; matrix size, 448 × 448 pixels. The patient was injected with gadolinium-diethylenetriamine pentaacetic acid (Gd-DTPA) in a dose of 0.1 mmol/kg at an amount of 15 mL, and the dynamic sequences were acquired at 6 timepoints, including 1 pre-enhanced and 5 post-enhanced (from 1 to 4.5 min after enhanced). In the imaging-subtype validation cohort, the T1-weighted images were obtained by GE scanners on a 1.5-Tesla magnet strength using a three-dimensional spoiled gradient-echo sequence with a gadolinium-based contrast agent. The spacing between slices of validation images ranged from 2 to 3 mm, the in-plane resolution ranged from 0.53 to 0.86 mm, the acquisition matrix was 256 × 192, and the flip angle was 10 degrees. The echo time and repetition time were not available in the validation cohort. The data used from the TCIA database in this study followed the Data Usage Policies and Restrictions of TCIA [26].

### 2.5. Tumor Segmentation

Firstly, to correct the motion during dynamic enhancement, the post-contrast images were registered to the pre-contrast images using the affine registration method. Then, the threshold segmentation method was used to obtain VOI masks of tumor lesions roughly from the subtracted images of the first enhanced sequences. Both image registration and segmentation were performed in the open-source software 3D Slicer. Then, two radiologists blinded to the clinical information, one with ten years and another with three years of breast imaging experience, corrected the masks manually and confirmed tumor masks consensually.

### 2.6. Voxel-Based PE and SER Map Calculation

In order to not just extract features from the MR images and explore more radiomics features by taking advantage of the DCE-MRI technique, we tried to define and calculate the voxel-based PE and SER maps of VOI based on the signal intensity of each voxel for each patient in this work, according to the Breast Imaging-Reporting and Data System (BI-RADS) and some previous studies [27,28,29]. The N4 bias correction algorithm was applied to avoid data heterogeneity bias in all the 3T-MR images [30]. Then, the voxel-based PE maps quantified the relative change in the signal intensity for each voxel before and after contrast enhancement; voxel-based SER maps can describe the comparison of the signal intensity for each voxel during the post-contrast period. In this study, DCE-MRI sequences at four timepoints, including pre-contrast, early, middle, and late post-contrast images, were used to calculate the PE or SER maps of VOI. The voxel-based early PE map for each sample was obtained by Equation (1):

(1) $$PE_{early}=100\times \frac{I_{early}-I_{pre}}{I_{pre}}$$

where $I_{early}$ is the signal intensity of each voxel in the early contrast, and $I_{pre}$ is the voxel initial intensity before contrast. The voxel-based SER maps of both middle and late enhancement were calculated by Equation (2):

(2) $$SER_{map}=\frac{I_{early\;}-I_{pre}}{I_{map}-I_{pre}}$$

where $I_{map}$ here is the signal intensity of each enhanced voxel in middle or late images. As mentioned above, the effects of motion between different timepoints were eliminated because the post-contrast images were already registered on the pre-contrast images. N4 bias correction and the calculation of PE and SER maps were performed in Python 3.5.2.

### 2.7. Image Preprocessing and Radiomics Feature Extraction

Before feature extraction, images were resampled to a uniform voxel spacing (1 mm × 1 mm × 1 mm) by the B-Spline method and were normalized as well as remapped in the histogram to fit within μ ± 3σ (μ: mean gray-level within the volume of segmentation; σ: gray-level standard deviation), as absolute signal intensity values were not necessarily comparable between scanners. Pyradiomics (version 2.2.0) was used to perform image normalization, resampling, and radiomics feature extraction [31]. According to the Image Biomarker Standardization Initiative (IBSI), 14 shape features, 18 first-order features, and 22 gray level co-occurrence matrix (GLCM) texture features were extracted from the pre-contrast MR images for each patient in this study. Similarly, 18 first-order features and 22 GLCM texture features were extracted from the early PE, middle SER, and late SER maps, respectively. In total, 174 radiomics features were calculated and used in this study. Image preprocessing and feature extraction were all performed in Python 3.5.2.

### 2.8. Identification and Analysis of Imaging Subtypes

The consensus clustering algorithm was used to identify intrinsic imaging subtypes in the discovery and validation cohorts [32]. The algorithm first subsamples both items and features from a data matrix and then clusters them into k classes. The process is repeated multiple times. The proportion of clustering runs in which two items are grouped in multiple repetitive clustering, named as pairwise consensus value, is calculated. The algorithm generates a consensus matrix for a given number of clusters k, which can provide a quantitative method to estimate the number of unsupervised classes in a dataset.

In this work, we scaled imaging features by z-score and performed a bootstrap procedure with 10,000 times 80% items and 80% features resampling using the partitioning around medoids algorithm with Spearman distance metric. By varying the number of clusters k from 2 to 8, we selected the optimal number of clusters, which generated the most stable consensus matrices and the most unambiguous cluster assignments across clustering runs. The optimal number of intrinsic unsupervised clusters was determined in the discovery cohort, and the same procedure was performed on the imaging-subtype validation cohort. Additionally, in-group proportion (IGP) statistical analysis was also used to demonstrate the reproducibility and reliability of novel subtypes [33]. IGP will be 100% if the clusters are identical between two datasets and will be 0% conversely.

One-way analysis of variance (ANOVA) and Tukey’s ‘Honest Significant Difference’ test was used to determine imaging features specific to each imaging subtype. The Student’s *t*-test was used to compare the significant differences in imaging features between two imaging subtypes. The 95% confidence interval (CI) was used to assess the differences in DCE-MRI features of each subtype. Principal component analysis (PCA) was performed to identify the contribution of imaging features to the novel subtypes.

We applied two-sided Pearson’s chi-squared test or Fisher’s exact test to test the independence of imaging subtypes from other clinical characteristics, including ER, PR, HER2, and Ki67, IHC subtypes, PAM50 subtypes, and clinical stages. The consensus clustering, IGP, and statistical analysis were performed in R 4.0.1.

To explore the relationship between imaging subtypes based on radiomics features and the parameters of the conventional pharmacokinetic model, we further calculated two key DCE-MRI quantitative parameters, K^trans^ and v_e_, in the discovery cohort using a population-averaged arterial input function (AIF)-based Tofts model [34,35]. K^trans^ was calculated by measuring the accumulation of Gd-DTPA-based contrast agent in the extracellular–extravascular space, and v_e_ was the fractional volume for extracellular space [35]. Statistical differences in K^trans^ and v_e_ between different imaging subtypes were analyzed using ANOVA and Student’s *t*-test. The estimation of pharmacokinetic parameters was implemented in 3D Slicer and statistical analysis was performed in R 4.0.1.

### 2.9. RNA Sequencing and Transcriptomic Analysis

Tumor tissue was collected from 199 patients in the discovery cohort, and the protocols of total RNA isolation and sequencing are described in the Supplementary Materials. Trimmomatic was used to control the sequencing quality with the following parameters: [LEADING:3 TRAILING:5 SLIDINGWINDOW: 4:15 MINLEN:60] [36]. RNA-seq reads were aligned to human genome 19 by STAR [37] and quantified by HTSeq-Count [38]. The expression values of 57,773 transcripts were quantified in the forms of both counts and FPKM (fragments per kilobase of exon per million reads mapped).

We identified PAM50 intrinsic subtypes for the patients in the discovery cohort by using genefu [39] and performed differential expression analysis between two imaging subtypes using DESeq2 [40]. Genes with adjusted *p*-values less than 0.05 were considered differentially expressed genes (DEGs). ANOVA was used to obtain subtype-specific genes among three DCE-MRI subtypes. Gene set enrichment analysis (GSEA) was conducted based on the DEGs to identify the Kyoto Encyclopedia of Genes and Genomes (KEGG) pathways that differ significantly between imaging subtypes with the cutoff of false discovery rate (FDR) less than 0.25 [41,42]. We used CIBERSORT with a BC-specific reference signature matrix developed from single-cell transcriptome data to characterize the relative proportion of the 15 cell types, including malignant, fibroblasts, and immune cells, in bulk RNA-seq data [43,44]. ANOVA was used to determine specific cell types for each imaging subtype. The Student’s t-test was used to compare the significant differences in cell proportions between two imaging subtypes. All analysis was performed in R 4.0.1.

### 2.10. Prognostic Analysis

Kaplan–Meier analysis with the log-rank test was used to assess the differences in disease-free survival (DFS) or overall survival (OS) among imaging subtypes. Due to the lack of image data in the external prognosis validation datasets, we established a classifier using the random forest with default parameters, except the number of trees was set to 2000, to predict imaging subtypes from gene expression profiles according to the imaging subtype-specific genes. The micro area under the receiver operating characteristic curve (AUC) for multi-classification was used to assess classifier performance. The analysis was performed in R 4.0.1.

## 3. Results

### 3.1. Identification and Validation of the DCE-MRI Subtypes

**Figure 2:**
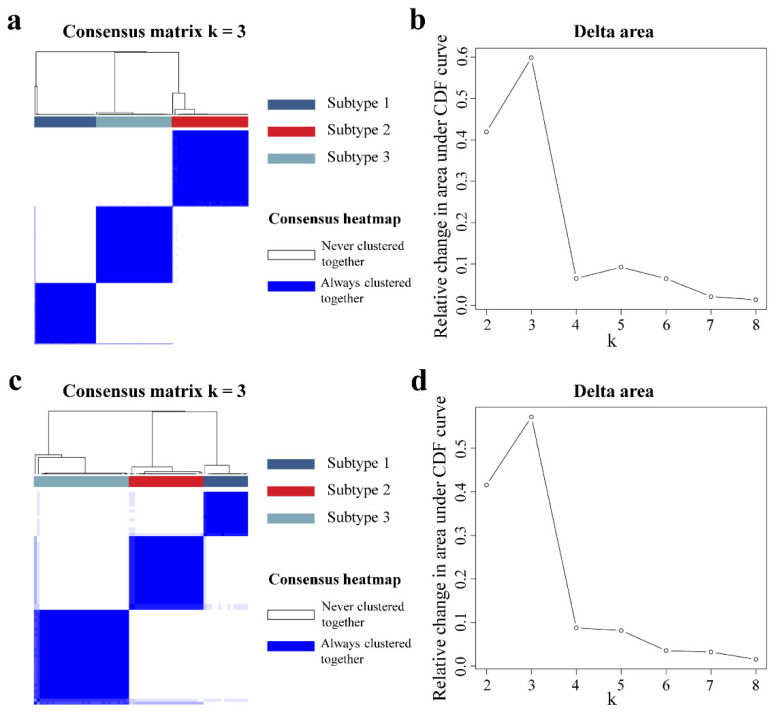
Unsupervised consensus clustering of DCE-MRI features. In the imaging-subtype discovery (**a**) and validation cohorts (**c**), the consensus matrix heatmaps for the optimal cluster number (k = 3) are displayed. Rows and columns of the consensus matrix were samples and values ranging from zero to one, indicating the two samples never clustered together or always clustered together, respectively. The dendrograms above the heatmaps indicate the samples ordering in three subtypes. The cluster number k varied from 2 to 8, and the optimal k could be determined to be 3 when yielding the largest relative change in area under the cumulative distribution function (CDF) curves for both the discovery (**b**) and validation cohorts (**d**).

The patient distribution of the two radiogenomics cohorts was not different except for the PAM50 molecular subtypes (Table 1), which indicated that there was no significant clinical bias for the patients. Then, we identified three de novo subtypes based on 174 DCE-MRI radiomics features using consensus clustering in the imaging-subtype discovery cohort (Figure 2a). The 3-cluster solution corresponded to the largest and optimal cluster number in the discovery cohort, which induced the least incremental change in the area under the cumulative distribution function (CDF) curve while keeping the maximal consensus within clusters and the minimal rate of ambiguity in cluster assignments (Figure 2b). Then, we independently applied the same analysis procedure on the imaging-subtype validation cohort, and, interestingly, we observed that samples in the validation cohort were also clustered into three optimal clusters based on the 174 features, similar to the discovery cohort (Figure 2c,d). The numbers of sample for imaging subtype 1, subtype 2 and subtype 3 were 50, 62, and 62 in the discovery cohort, respectively, as well as 15, 25, and 32 in the validation cohort. The reproducibility of the three imaging subtypes across both cohorts was evaluated by using IGP, resulting in values of 90.91%, 94%, and 90.91% for each subtype in the validation cohort. In short, the three DCE-MRI subtypes identified in the discovery cohort were validated in the validation cohort, indicating that these subtypes can reflect the intrinsic image heterogeneity of BC.

### 3.2. Imaging Characteristics of the DCE-MRI Subtypes

**Figure 3:**
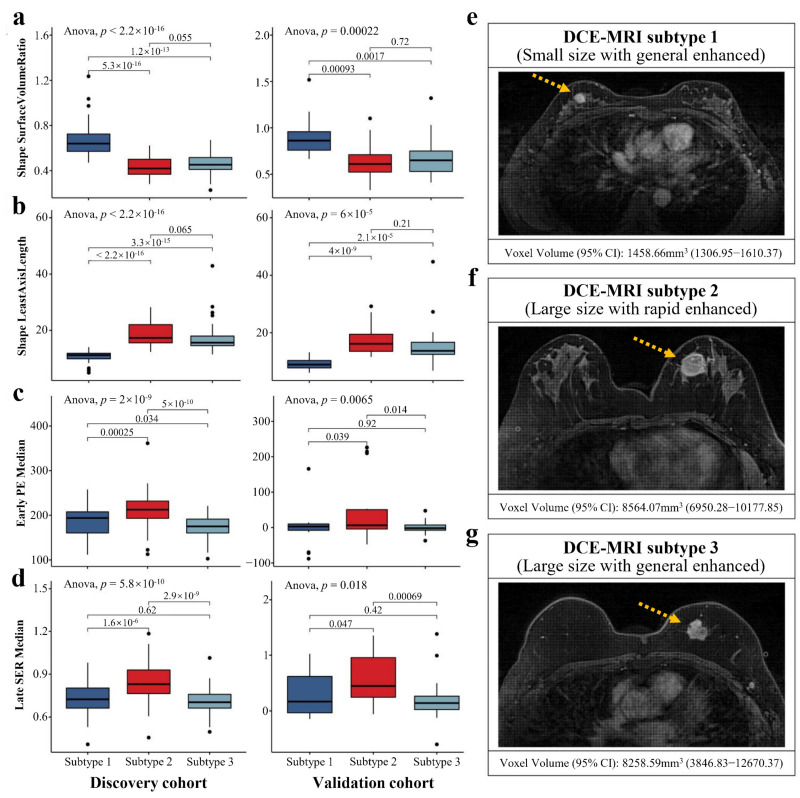
Significant differences in tumor sizes and enhanced patterns shaped the imaging subtypes. Three subtypes were significantly associated with four representative quantitative imaging features in both cohorts, including surface volume ratio (**a**), least axis length (**b**), early PE map median (**c**), and late SER map median (**d**). ANOVA was used to identify the differences in imaging features among three subtypes, and Student’s *t*-test was used for the comparison between two subtypes. The mean values with 95% CI of the selected imaging feature and the representative sample from the discovery cohort for each imaging subtype are illustrated in (**e**–**g**) (yellow arrows point to the tumors).

We examined the difference in imaging features among the DCE-MRI subtypes. The *p*-values of ANOVA for each imaging feature in the discovery and validation cohorts are listed in Table S1. The PCA results indicated that the 58 important imaging features constitute the heterogeneity of the three imaging subtypes (Figure S2). Remarkably, we found that tumor shape features, such as surface volume ratio and least axis length, showed extremely significant differences among the three imaging subtypes in both cohorts (*p*-value < 0.001, Figure 3a,b). In the discovery cohort, subtype 1 typically had smaller tumor size (mean voxel volume [95% CI], 1458.66 mm^3^ [1306.95–1610.37]) compared to subtypes 2 and 3 (*p*-value < 0.01), while there was no significant difference in tumor size between subtypes 2 (mean voxel volume [95% CI], 8564.07 mm^3^ [6950.28–10,177.85]) and 3 (mean voxel volume [95% CI], 8258.59 mm^3^ [3846.83–12,670.37]). The imaging features that can reflect dynamic enhanced characteristics of the tumor, including the medians of early PE and late SER maps, demonstrated highly significant differences among imaging subtypes in both cohorts (*p*-value < 0.05, Figure 3c,d). We found that during dynamic contrast enhancement, BC samples of subtype 2 showed a pattern of rapid enhancement, while subtypes 1 and 3 showed a generally enhanced pattern. Based on the clinical imaging characteristics, we respectively defined subtype 1 as small size with generally enhanced pattern tumor (Figure 3e), subtype 2 as large size with rapidly enhanced pattern tumor (Figure 3f), and subtype 3 as large size with generally enhanced pattern tumor (Figure 3g).

It was interesting to note that when analyzing the differences in pharmacokinetic parameters between the three imaging subtypes, we found significant differences in K^trans^ among the imaging subtypes (*p*-value < 0.01, Figure S3a). Compared to subtypes 1 and 3, BC patients with subtype 2 had significantly higher K^trans^ values (mean value, 0.1867 min^−1^), which was consistent with the findings based on the rapid enhancement pattern defined by quantitative radiomics. Although v_e_ did not differ significantly among the three imaging subtypes, it was different between subtypes 1 and 2 (*p*-value < 0.05, Figure S3b).

### 3.3. Distinct Prognostic Outcomes of the DCE-MRI Subtypes

**Figure 4:**
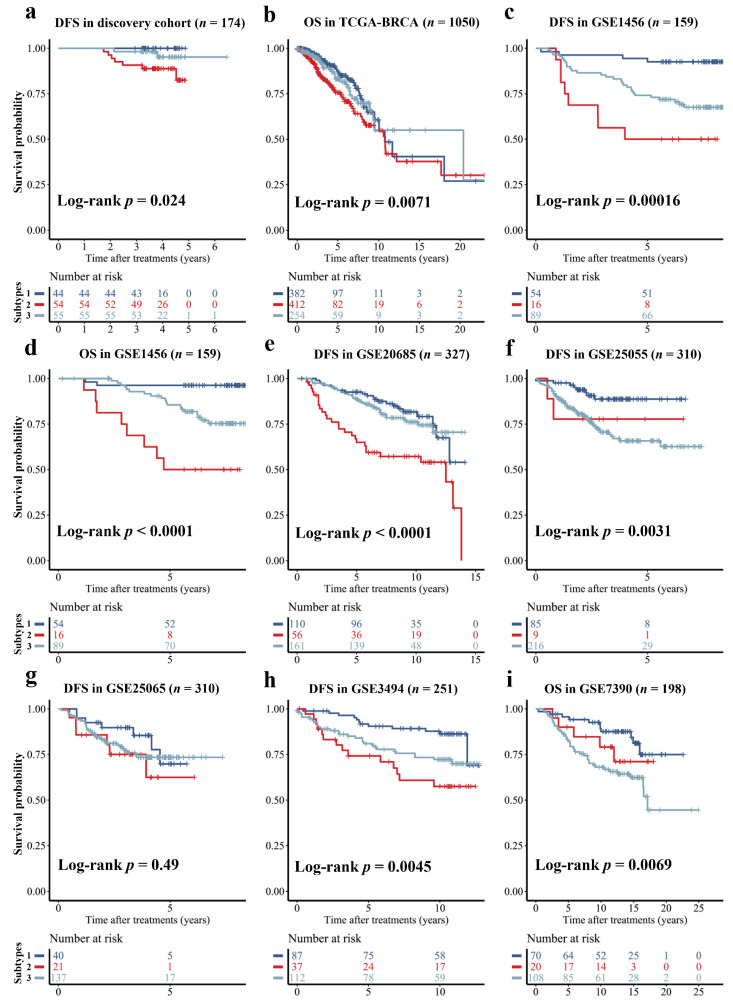
Prognosis of DFS or OS stratified by the DCE-MRI subtypes. The Kaplan-Meier curve was plotted for the stratified samples by three imaging subtypes in the discovery cohort (**a**), and evaluated in seven external datasets without imaging data but by using a gene-profile-based imaging subtypes classifier to determine the imaging subtypes (**b**–**i**).

We found significant DFS differences among the three DCE-MRI subtypes in the discovery cohort (*p*-value < 0.05, Figure 4a). To assess the prognostic ability of imaging subtypes further, we selected 993 subtype-specific genes by one-way ANOVA in the discovery cohort and established the classifier using random forest to predict imaging subtypes from gene expression profiles. This classifier achieved a moderate multi-classification AUC with 0.6 in the independent validation cohort for the prediction of imaging subtypes. Then, we applied the classifier to identify imaging subtypes in six external prognosis validation datasets which only had gene expression profiles, and found significantly different prognostic outcomes, including DFS and OS, for the imaging subtypes (*p*-value < 0.01, Figure 4). Notably, the pattern of outcome in external prognosis validation datasets was consistent with the imaging-subtype discovery cohort, with subtype 1 showing a favorable prognosis, subtype 2 having the worst prognosis, and subtype 3 displaying an intermediate prognosis.

### 3.4. Associations with the Established BC Subtypes and Clinical Stages

**Figure 5:**
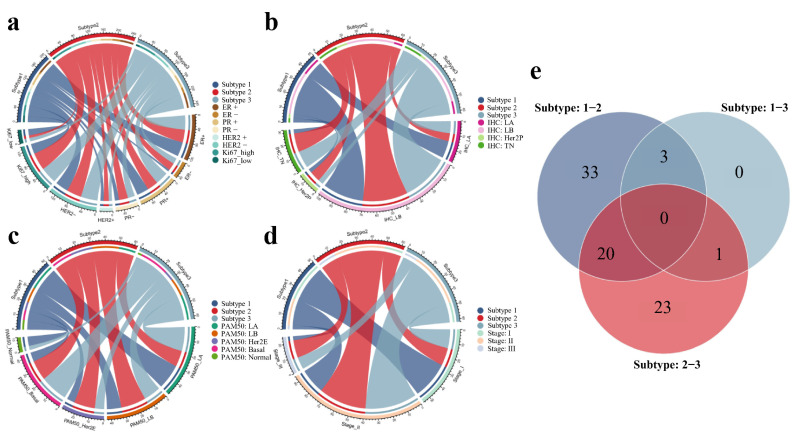
Associations of the DCE-MRI subtypes with clinical and pathway characteristics. The chord diagrams between three imaging subtypes and clinical IHC receptors (**a**), IHC-based subtypes (**b**), PAM50 molecular subtypes (**c**), and clinical stages (**d**) in the discovery cohort were described. Venn diagram of the differentially enriched KEGG pathways between two of three imaging subtypes (**e**).

Associations between the novel imaging subtypes and established BC subtypes or tumor stages in the discovery cohort are detailed in Table 2. We noticed imaging subtypes were significantly associated with Ki67 status, PAM50 intrinsic molecular subtypes, and tumor stages (*p*-value < 0.01). In the discovery cohort, 77.2% of samples with high Ki67 expression belonged to subtype 2 or subtype 3, whereas subtype 1 was more likely to have a low Ki67 expression (*p*-value = 0.004, Figure 5a). We also observed that 90% of TN samples belonged to subtype 2 or subtype 3 (Figure 5b), and a similar distribution (83.7%) was revealed in basal-like cases (*p*-value = 0.005, Figure 5c). Remarkably, we found that the proportion of favorable prognosis for subtype 1 became lower as the tumor stage increased, while subtypes 2 and 3 with worse prognosis were more prevalent in higher stage (*p*-value < 0.001, Figure 5d). High Ki67 expression, TN or basal-like subtype and high-grade tumor stage were usually regarded as the potential risk factors for clinical assessment of BC malignancy and poor prognosis. The distinct patterns of clinical outcome of imaging subtypes were consistent with the associations between the three subtypes and these risk factors.

### 3.5. The Differences of Molecular and Microenvironment Characteristics among Imaging Subtypes

**Figure 6:**
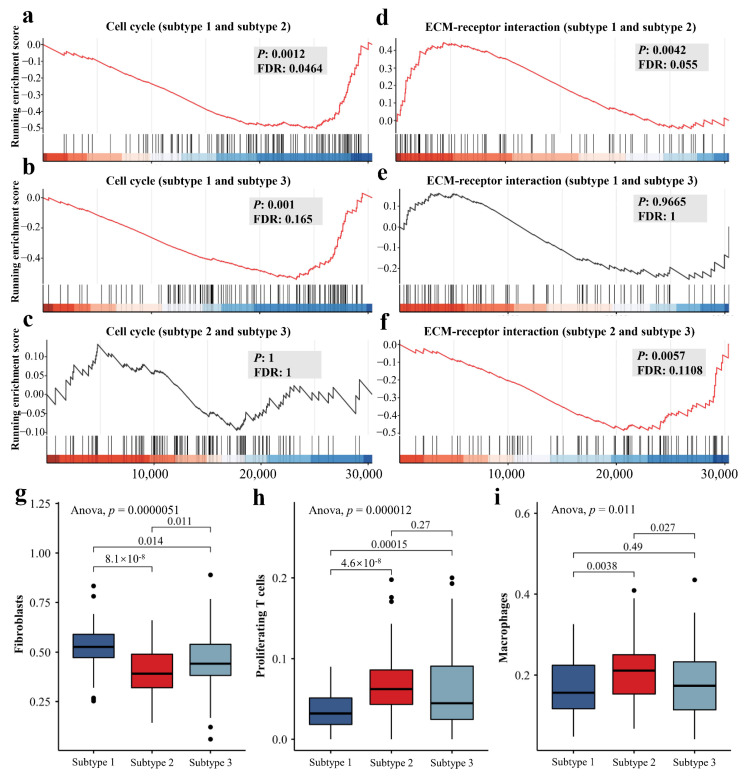
Important enriched KEGG pathways and tumor microenvironment characteristics of different DCE-MRI subtypes. Detailed differences in representative transcriptional behavior among three subtypes including cell cycle (**a**–**c**) and ECM-receptor interaction (**d**–**f**) are illustrated. The boxplots for the fractions of cancer-associated fibroblasts (**g**), proliferating T cells (**h**) and macrophages (**i**) among imaging subtypes in both discovery and validation cohorts are displayed. ANOVA was used to identify the differences in cellular fractions among three subtypes, and Student’s t-test was used for the comparison between two subtypes.

We identified 56 different enriched KEGG pathways between subtypes 1 and 2, 4 pathways between subtypes 1 and 3, and 44 between subtypes 2 and 3, respectively, by gene differential expression analysis and GSEA (FDR < 0.25, Tables S2–S4). These imaging subtype-associated KEGG pathways included cell cycle, ECM-receptor interaction, Hedgehog signaling pathway, proteoglycans in cancer, PI3K-Akt signaling pathway, Ras signaling pathway, and breast cancer. Interestingly, based on the heterogeneity of molecular pathways, subtype 2 was distant from the other two subtypes, while subtypes 1 and 3 were much closer (Figure 5e). Compared to subtype 1, the cell cycle was significantly enriched at the bottom in both subtypes 2 and 3 (FDR = 0.0464 and 0.165, Figure 6a,b), while there was no difference between subtype 2 and subtype 3 (Figure 6c). We also observed that ECM-receptor interaction was significantly different between imaging subtype 2 and subtypes 1 and 3 (FDR = 0.055 and 0.1108, Figure 6d,f), while there was no difference between subtype 1 and subtype 3 (Figure 6e). Notably, the enrichment trend of ECM-receptor interaction in subtype 2 with a worse prognosis was similar, compared to the subtypes with slightly better prognosis. The extracellular matrix (ECM) is an essential component of the tumor microenvironment and plays an important role in tumorigenesis, proliferation, and metastasis. Therefore, we further estimated the abundance of 15 cell types in the samples from both discovery and validation cohorts, including malignant, fibroblasts, and immune cells. Significant differences in the abundance of fibroblasts, proliferating T cells and macrophages were revealed among three imaging subtypes in the discovery cohort (*p*-value < 0.05, Figure 6g–i, Table 3), and interestingly similar trends were also observed in the independent validation cohort (Figure S4).

## 4. Discussion

In this study, we identified and validated three distinct BC imaging subtypes by using DCE-MRI features of tumor lesions in two cohorts. The novel BC subtypes had significantly different tumor sizes and imaging enhancement patterns, as well as clinical outcomes. Importantly, the independent prognostic value of imaging subtypes was demonstrated in our discovery cohort, and by using a gene expression-based subtype classifier, we displayed this value in six external datasets. We further discovered that the imaging subtypes significantly correlated with Ki67, PAM50 subtypes and clinical stages, and revealed the biological mechanisms underlying this heterogeneity. Three subtypes not only differed significantly in their transcriptional activities such as cell cycle and ECM-receptor interaction KEGG pathways, but also had different microenvironment characteristics, particularly in the abundance of fibroblasts, proliferating T-cells, and macrophages. In summary, we revealed BC heterogeneity from a new perspective and uncovered the possible biological mechanisms, which may be useful for BC clinical decision making.

Tumor size and enhancement patterns were the most representative imaging differences in our imaging subtypes. As a critical determinant of clinical outcome and staging system for BC, tumor size is strongly correlated with prognosis, and a larger BC usually suggests a worse outcome. A tumor with a rapid uptake in the early-enhanced process of DCE-MRI and a quick washout in the later part of enhancement suggests more malignancy [45,46,47]. Imaging subtype 2 with larger tumor size, early enhanced rapid pattern and quickly reduced late pattern (Figure 3b–d and Figure S3a) were significantly associated with poorer DFS of BC (Figure 4a). Conversely, subtype 1 had the best clinical outcome, indicating that our findings were consistent with previous studies. More importantly, the three subtypes displayed high reproducibility (IGP over 90%) in the two cohorts (Figure 2) and had similar prognostic trends in multiple external datasets (Figure 4).

The heterogeneity of imaging features may arise from the differences in biological mechanisms. We identified 80 out of 186 KEGG pathways associated with the imaging subtypes, suggesting a link between DCE-MRI features and transcriptional activities. These pathways, including cell cycle, ECM-receptor interaction, PI3K-Akt signaling pathway and homologous recombination, fall into several broad categories involving a variety of BC-related biological activities such as cellular processes, metabolism, and genetic and environmental information processing. We found biologically significant differences between imaging subtype 2 and other subtypes, while subtypes 1 and 3 were more similar. The tail-end enrichment of cell cycle and a higher proportion of Ki67 low-expressing patients, implied a reduced capacity for tumor growth and proliferation in subtype 1, resulting in smaller tumor size and favorable prognosis. Profound changes in cellular metabolism were usually a hallmark of malignancy, and subtype 2, which had the worst prognosis displayed remarkable metabolic disorders compared to other subtypes [48,49,50]. For example, as a key oxidative enzyme, cytochrome P450 can metabolize many carcinogens and anticancer drugs in BC [51]. We discovered that two cytochrome P450-related pathways (metabolism of xenobiotics by cytochrome P450 and drug metabolism-cytochrome P450) were specific to subtype 2. Furthermore, although both subtypes 2 and 3 had larger tumor size (Figure 3a,b), their enhancement patterns were significantly different (Figure 3c,d and Figure S3a). The possible biological mechanisms for this result could be explained by the fact that subtypes 2 and 3 displayed no difference in cell cycle but had significant differences in many pathways, such as ECM-receptor interaction and some metabolic pathways. ECM is an essential component of the tumor microenvironment and plays an important role in regulating BC progression and metastasis, which might affect the process of enhancement imaging [52,53,54,55]. Our work may provide a non-invasive perspective on the biology of BC.

Differences in the tumor immune microenvironment emerged in the imaging subtypes. Subtype 2 exhibited a lower proportion of fibroblasts and a higher proportion of proliferating T cells and macrophages. Cancer-associated fibroblasts (CAFs) are not only important components of the BC microenvironment and are essential in tumor growth and development, but also have complex phenotypes and functional heterogeneity [56,57,58,59]. T cell proliferation is closely related to immune response, treatment resistance, and prognosis of BC [60,61,62]. Macrophages are important players in the extracellular matrix activity of breast tumors and a high fraction of macrophages usually suggests a poorer prognosis [63,64].

Differing from most radiogenomics studies that focused on the association of imaging features with gene expression profiles, we uncovered the heterogeneity of BC from the novel perspective of radiomics, and further revealed the differences of clinical outcome and biological activities for imaging subtypes. Although some previous works have used similar approaches to identify potential imaging subtypes of glioblastoma and BC based on MRI data [65,66], our work still has some strengths, especially compared to the work of Wu et al. [66]. Firstly, we identified three imaging subtypes of BC in a larger dataset and achieved high cluster reproducibility in an independent multi-center validation cohort (all of our IGPs were greater than 90%, whereas only one IGP of Wu et al. [66] was greater than 90%). Secondly, the determination of breast background parenchymal tissue is a challenging task in clinical practice, whereas the segmentation of tumor lesions is much easier. We predicted the prognosis well using only the information of tumor lesions, which is more convenient for clinical applications. Additionally, we highlighted the clinical evidence of DCE-MRI features as non-invasive biomarkers. In the radiomics discovery cohort, the mean voxel volume with 95% CI of imaging subtype 1 was 1458.66 mm^3^ [1306.95–1610.37], and larger tumor sizes were observed in subtype 2 (8564.07 mm^3^ [6950.28–10,177.85]) and subtype 3 (8258.59 mm^3^ [3846.83–12,670.37]). Finally, we not only revealed the differences in biological functions of imaging subtypes, but also further analyzed the associations of cellular fractions with imaging subtypes, although the abundance of cells was estimated from bulk RNA-seq data. It was noteworthy that we used a BC-specific expression matrix based on single-cell data to estimate cellular fractions in bulk RNA-seq data, which indicated that the tumor microenvironment was disease-specific. There were some limitations in our work. Studies supporting the possible influence of biological activity on imaging features, and whether this correlation is causal, still need to be verified in conjunction with biological experiments. The enhancement patterns of DCE-MR imaging that define imaging subtypes need to be analyzed for differences in vascular permeability based on histopathology data to further validate our findings. The relationship between the complex characteristics of the tumor microenvironment and imaging subtypes may require deeper mining in combination with single-cell RNA-seq data. Additionally, the gene-profile-based classifier of DCE-MRI subtypes did not perform very well, and should be improved based on more radiogenomics cohorts of BC in the future, and the robustness of the prognostic value of the imaging features needs to be further evaluated in larger datasets for clinical applicability.

## 5. Conclusions

In this study, three novel BC subtypes were identified and validated based on the radiomics features from tumor lesions in two independent DCE-MRI cohorts. The imaging subtypes showed distinct clinical outcomes, molecular characteristics, and cellular fractions in the tumor microenvironment. Our work may provide a potential radiomics biomarker for the precision medicine of BC.

## Figures and Tables

**Table 1:** Demographics of BC patients in the imaging-subtype discovery and validation cohorts. The detailed clinical, imaging, and molecular data for the discovery and validation of radiomics cohorts. The patient distribution of the two cohorts was not different except for the PAM50 molecular subtypes.

Characteristics	Discovery Cohort (*n* = 174)	Validation Cohort (*n* = 72)	*p*-Value
Age, mean (SD)	≤50 years:95/>50 years:79; 49.78 years (9.99)	≤50 years:30/>50 years:42; 53.96 years (11.75)	0.088 ^a^
Histopathology type			0.069 ^a^
Ductal	156	71	
Lobular	4	0	
Mixed	12	0	
Other	2	1	
BI-RADS			NA
Category 3	1	NA	
Category 4	61	NA	
Category 5	103	NA	
Category 6	9	NA	
IHC receptors			
ER status	P:127/N:47	P:61/N:11	0.071 ^a^
PR status	P:111/N:63	P:55/N:17	0.077 ^a^
HER2 status	P:36/N:138	P:14 / N:37/NA:21	0.407 ^a^
Ki67 status	high:136/low:38	NA	NA
IHC-based subtype			
Luminal-A	28	NA	NA
Luminal-B	101	NA	NA
HER2-positive	15	NA	NA
Triple-negative	30	NA	NA
PAM50 subtype			<0.001 ^a^
Luminal-A	49	44	
Luminal-B	43	9	
HER2-Enriched	29	5	
Basal-like	43	10	
Normal-like	10	4	
Pathological stage			0.267 ^a^
Stage I	55	17	
Stage II	94	47	
Stage III	25	8	

Note: unless otherwise indicated, data are the number of patients or the *p*-value of the statistical test. ^a^ *p*-value for the two-sided Pearson’s chi-squared test. SD, standard deviation; P, positive receptor status; N, negative. BI-RADS, Breast Imaging-Reporting and Data System. NA: not available.

**Table 2:** Differences of established subtypes and clinical stages for DCE-MRI subtypes in the imaging-subtype discovery cohort. The relationships between three imaging subtypes and established subtypes of BC, including clinical receptor-based, IHC-based and PAM50-based subtypes, as well as the clinical stages, were calculated.

Factors	Subtype 1 (*n* = 50)	Subtype 2 (*n* = 62)	Subtype 3 (*n* = 62)	*p*-Value
ER status				0.011 ^a^
ER positive	44	39	44	
ER negative	6	23	18	
PR status				0.109 ^a^
PR positive	37	34	40	
PR negative	13	28	22	
HER2 status				0.409 ^a^
HER2 positive	10	16	10	
HER2 negative	40	46	52	
Ki67 status				0.004 ^a^
Ki67 high	31	54	51	
Ki67 low	19	8	11	
IHC-based subtype				0.051 ^b^
Luminal-A	13	6	9	
Luminal-B	31	34	36	
HER2-positive	3	8	4	
Triple-negative	3	14	13	
PAM50 subtype				0.005 ^b^
Luminal-A	19	12	18	
Luminal-B	11	19	13	
HER2-Enriched	6	16	7	
Basal-like	7	15	21	
Normal-like	7	0	3	
Pathological stage				<0.001 ^a^
Stage I	28	16	14	
Stage II	19	36	39	
Stage III	3	13	9	

Note: unless otherwise indicated, data are number of patients or the *p*-value of the statistical test. ^a^ *p*-value for the two-sided Pearson’s chi-squared test, ^b^ *p*-value for the two-sided Fisher’s exact test.

**Table 3:** Immune microenvironment cellular fraction differences of imaging subtypes in the imaging-subtype discovery cohort. A BC-specific reference signature matrix developed from single-cell transcriptome data was used to estimate the relative proportion of 15 cell types in the bulk RNA-seq data. Then, the differences in the cellular fraction of imaging subtypes were assessed.

Cell Types	*p*-Value	Subtype 2-1	Subtype 3-1	Subtype 3-2
Malignant cells	0.166	0.141	0.517	0.67
Fibroblasts	5.11 × 10^−6^ *	2.58 × 10^−6^ *	0.031^*^	0.021 *
Proliferating T cells	1.18 × 10^−5^ *	1.06 × 10^−5^ *	0.001^*^	0.416
Cytotoxic T cells	NaN	NaN	NaN	NaN
Regulatory T cells	0.738	0.751	0.795	0.996
Naive-like T cells	0.408	1	0.504	0.464
Natural killer cells	0.408	1	0.504	0.464
Neutrophils	0.697	0.848	0.969	0.683
Plasma cells	0.241	0.218	0.759	0.565
Dendritic cells	0.408	0.504	1	0.464
Macrophages	0.011 *	0.013 *	0.773	0.059
Monocytes	0.8	0.911	0.976	0.788
Mast cells	0.408	0.504	1	0.464
B cells	0.704	0.896	0.679	0.91
Transitional T cells	0.884	0.892	0.996	0.923

Note: unless otherwise indicated, data are the *p*-value of ANOVA or Tukey’s test. * *p*-value for *p*-value < 0.05. NaN: Not a Number.

## Data Availability

The RNA-seq data of the imaging-subtype discovery cohort are available at https://ngdc.cncb.ac.cn/bioproject/ (BioProject number: PRJCA005965, GSA-Human number: HRA001100, accessed on 1 October 2019). The TCGA-BRCA gene expression data are available at https://portal.gdc.cancer.gov/projects/TCGA-BRCA (accessed on 31 January 2019) and the corresponding DCE-MRI data are available at https://wiki.cancerimagingarchive.net/display/Public/TCGA-BRCA (accessed on 20 October 2019). Further information and other data that support the findings of this study are available from the corresponding author upon reasonable request.

## Supplementary Materials

The following supporting information can be downloaded at: https://www.mdpi.com/article/10.3390/cancers14225507/s1, Figure S1: The inclusion and exclusion criteria of BC patients for radiomics cohorts. Figure S2: PCA of significantly different imaging features in the imaging-subtype discovery cohort. Figure S3: Differences in pharmacokinetic parameters of patients with different imaging subtypes in the imaging-subtype discovery cohort. Figure S4: Distinct cellular fractions in the tumor microenvironment of different imaging subtypes in the imaging-subtype validation cohort. Table S1: The *p*-values of ANOVA for DCE-MR features of three imaging subtypes in the imaging-subtype discovery and validation cohorts. Table S2: Differentially enriched KEGG pathways between imaging subtypes 1 and 2 in the imaging-subtype discovery cohort. Table S3: Differentially enriched KEGG pathways between imaging subtypes 1 and 3 in the imaging-subtype discovery cohort. Table S4: Differentially enriched KEGG pathways between imaging subtypes 2 and 3 in the imaging-subtype discovery cohort.
