# Supplementary material for: Unsupervised Analysis Based on DCE-MRI Radiomics Features Revealed Three Novel Breast Cancer Subtypes with Distinct Clinical Outcomes and Biological Characteristics
Source: Cancers (Basel). 2022 Nov 9;14(22):5507. doi: 10.3390/cancers14225507 (PMC9688868; doi:10.3390/cancers14225507)
Supplement: Supplementary file 1 [file cancers-14-05507-s001.zip › cancers-1966846-supplementary.pdf]

Supplementary Materials of

Unsupervised Analysis Based on DCE-MRI Radiomics Features Revealed Three Novel Breast Cancer Subtypes with Distinct Clinical Outcomes and Biological Characteristics

Wenlong Ming, Fuyu Li, Yanhui Zhu, Yunfei Bai, Wanjun Gu, Yun Liu, Xiaoan Liu, Xiao Sun * and Hongde Liu *

* Correspondence: xsun@seu.edu.cn (X.S.); liuhongde@seu.edu.cn (H.L.)

Figure S1. The inclusion and exclusion criteria of BC patients for radiomics cohorts.

Figure S2. PCA of significantly different imaging features in the imaging-subtype discovery cohort.

Figure S3. Differences in pharmacokinetic parameters of patients with different imaging subtypes in the imaging-subtype discovery cohort.

Figure S4. Distinct cellular fractions in tumor microenvironment of different imaging subtypes in the imaging-subtype validation cohort.

Table S1. The *P*-values of ANOVA for DCE-MR features of three imaging subtypes in the imaging-subtype discovery and validation cohorts.

Table S2. Differentially enriched KEGG pathways between imaging subtypes 1 and 2 in the imaging-subtype discovery cohort.

Table S3. Differentially enriched KEGG pathways between imaging subtypes 1 and 3 in the imaging-subtype discovery cohort.

Table S4. Differentially enriched KEGG pathways between imaging subtypes 2 and 3 in the imaging-subtype discovery cohort.

Supplementary Methods.

Figures

Radiomics cohorts collection and inclusion

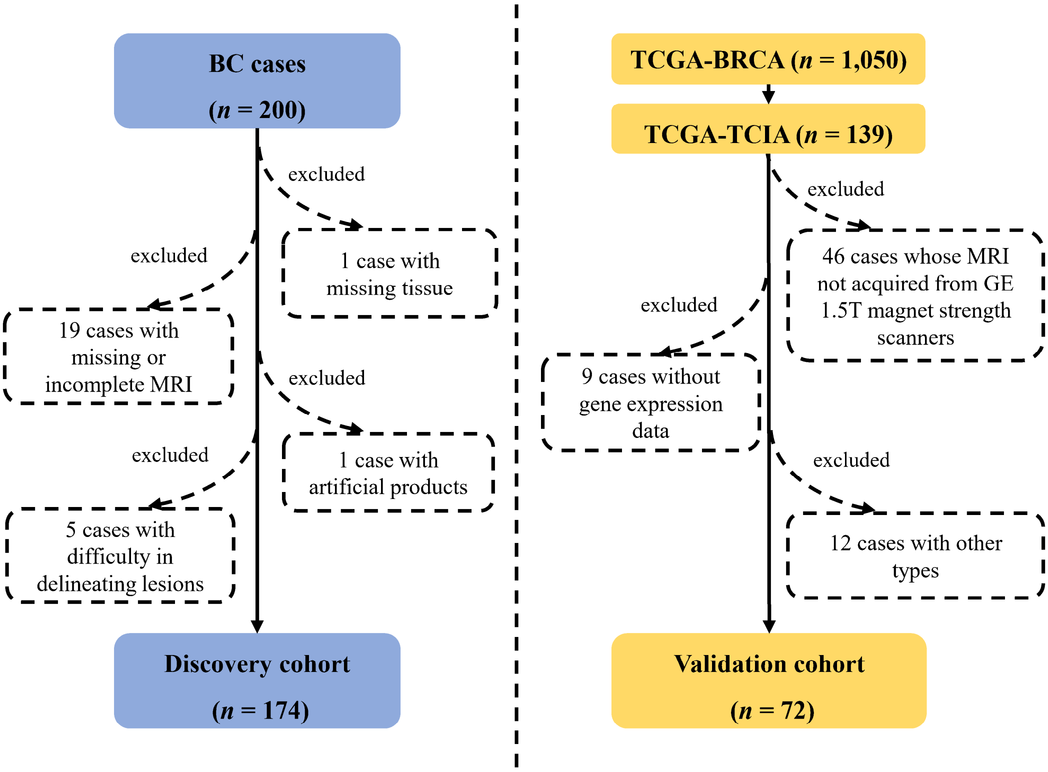

Figure S1. The inclusion and exclusion criteria of BC patients for radiomics cohorts. In this study, we enrolled 246 BC patients for radiomics analysis totally.

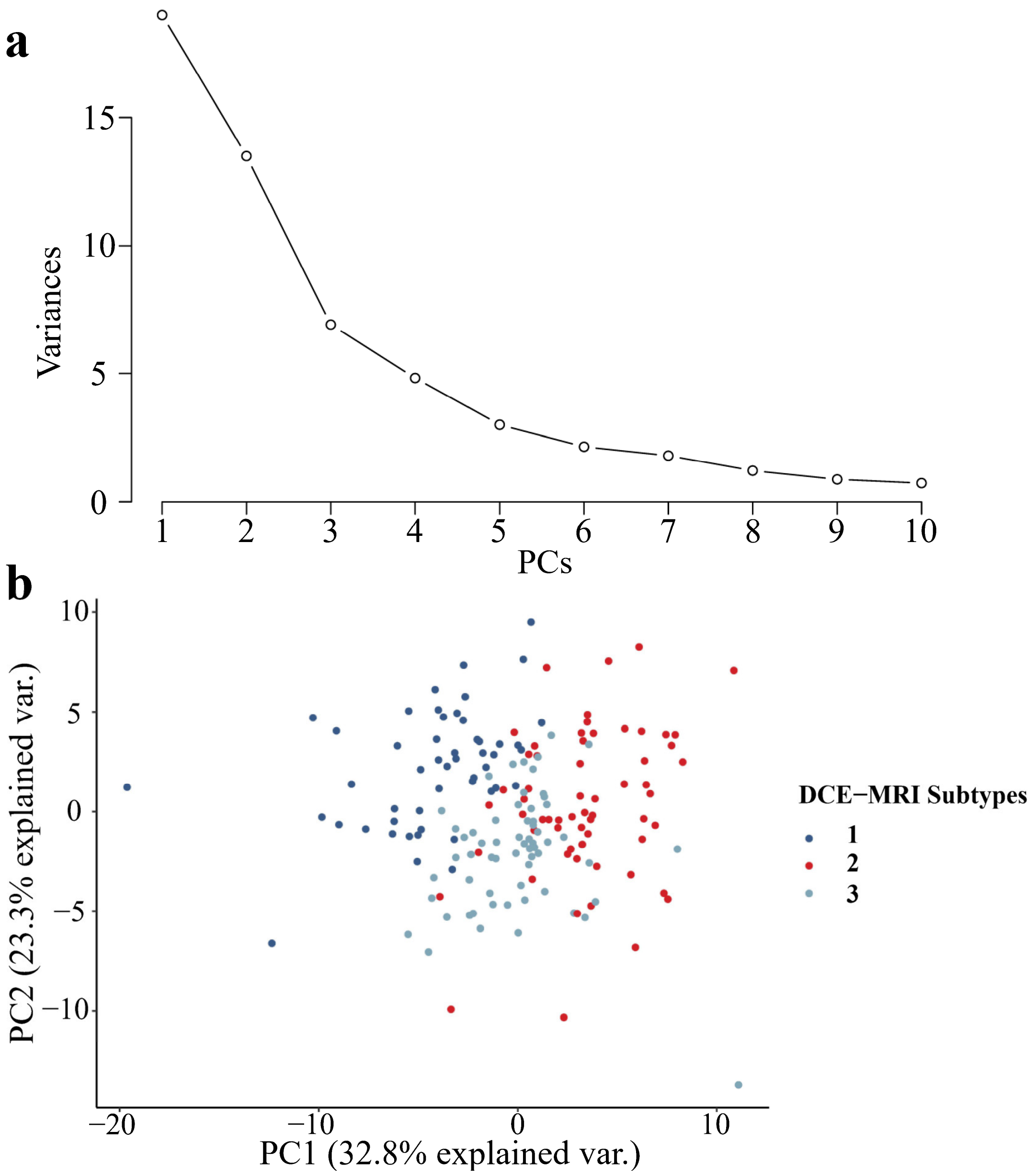

Figure S2. PCA of significantly different imaging features in the imaging-subtype discovery cohort. We filtered 58 imaging features with the cutoff of ANOVA p-value < 0.0001 and performed PCA. The variances of the top 10 principal components (PCs) were displayed (**a**), and the first two PCs could well stratify patients into three imaging subtypes (**b**).

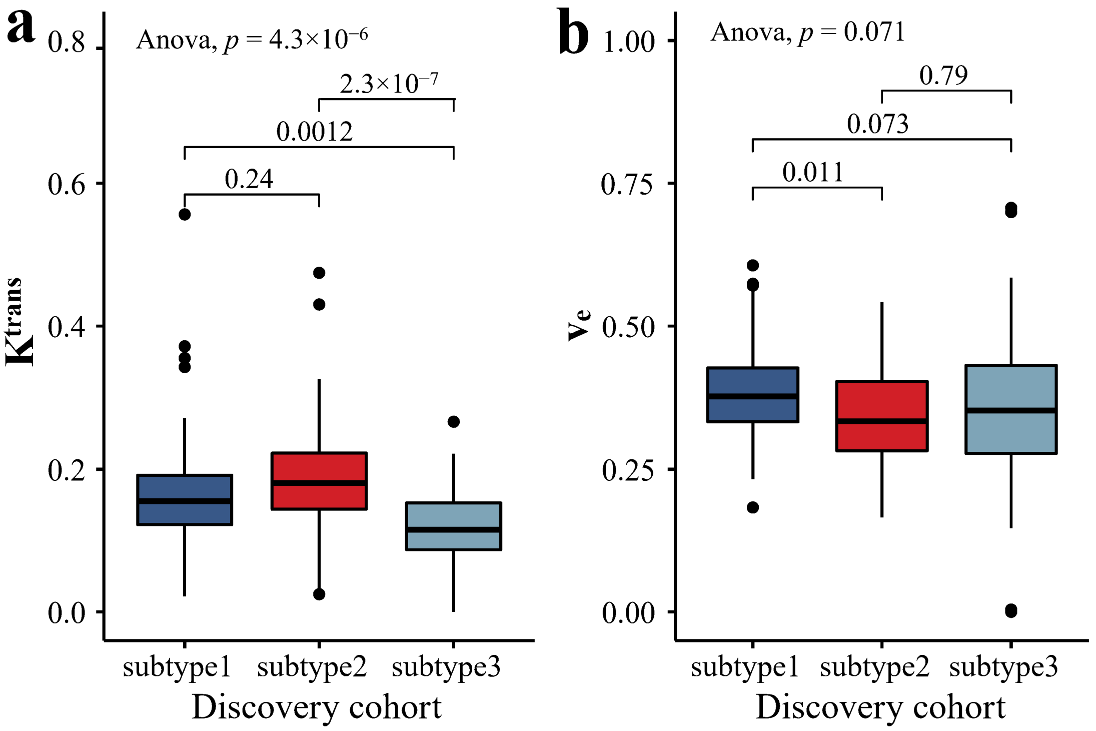

Figure S3. Differences in pharmacokinetic parameters of patients with different imaging subtypes in the imaging-subtype discovery cohort. The boxplots for the K^{trans} (**a**) and v_e (**b**) among three imaging subtypes in the discovery cohort were displayed. ANOVA was used to identify the differences in pharmacokinetic parameters among the three subtypes, and the Student's *t*-test was used for the comparison between the two subtypes.

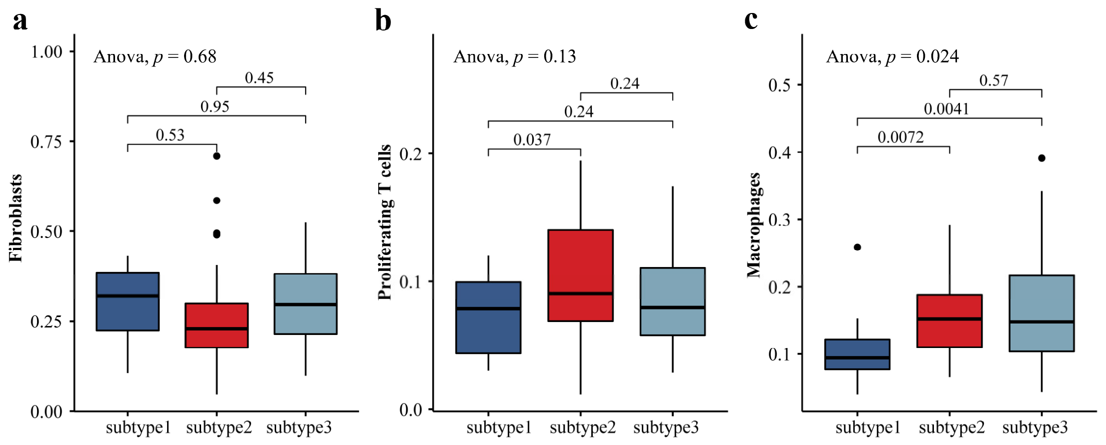

Figure S4. Distinct cellular fractions in tumor microenvironment of different imaging subtypes in the imaging-subtype validation cohort. The boxplots for the fractions of cancer-associated fibroblasts (**a**), proliferating T cells (**b**) and macrophages (**c**) among three imaging subtypes in the validation cohort were displayed. ANOVA was used to identify the differences of cellular fraction among the three subtypes, and the Student's *t*-test was used for the comparison between the two subtypes.

Tables

Table S1. **The *P*-values of ANOVA for DCE-MR features of three imaging subtypes in the imaging-subtype discovery and validation cohorts.** A number of 174 quantitative DCE-MR imaging features from tumor lesion region were used in this study, including 14 shape features, 18 first-order features, and 22 GLCM texture features from the pre-contrast MR images (PreMRI), 18 first-order features and 22 GLCM texture features from the voxel-based early PE map (EarlyPE), 18 first-order features and 22 GLCM texture features from the voxel-based middle SER map (MiddleSER), as well as 18 first-order features and 22 GLCM texture features from the voxel-based late SER map (LateSER).

DCE-MRI features	Discovery	Validation
Shape_VoxelVolume	1.13×10^{-3}	1.75×10^{-1}
Shape_SurfaceVolumeRatio	6.96×10^{-25}	2.19×10^{-4}
Shape_SurfaceArea	6.88×10^{-5}	9.97×10^{-2}
Shape_Sphericity	4.46×10^{-4}	1.07×10^{-2}
Shape_MinorAxisLength	1.65×10^{-15}	2.48×10^{-4}
Shape_MeshVolume	1.15×10^{-3}	1.78×10^{-1}
Shape_Maximum3DDiameter	1.36×10^{-13}	4.77×10^{-4}
Shape_Maximum2DDiameterSlice	5.71×10^{-16}	2.59×10^{-4}
Shape_Maximum2DDiameterRow	7.14×10^{-16}	4.23×10^{-4}
Shape_Maximum2DDiameterColumn	9.06×10^{-12}	9.87×10^{-5}
Shape_MajorAxisLength	9.35×10^{-14}	5.74×10^{-4}
Shape_LeastAxisLength	3.11×10^{-21}	6.05×10^{-5}
Shape_Flatness	7.03×10^{-1}	8.49×10^{-1}
Shape_Elongation	5.68×10^{-1}	9.54×10^{-1}
PreMRI_glcm_SumSquares	1.19×10^{-2}	2.11×10^{-2}
PreMRI_glcm_SumEntropy	2.14×10^{-2}	1.20×10^{-5}
PreMRI_glcm_MaximumProbability	1.18×10^{-2}	1.13×10^{-3}
PreMRI_glcm_JointEntropy	8.40×10^{-3}	3.85×10^{-6}
PreMRI_glcm_JointEnergy	3.02×10^{-2}	4.98×10^{-3}
PreMRI_glcm_JointAverage	1.46×10^{-9}	1.20×10^{-6}
PreMRI_glcm_InverseVariance	4.21×10^{-2}	9.61×10^{-2}
PreMRI_glcm_Imc2	6.34×10^{-3}	2.93×10^{-3}

PreMRI_glcm_Imc1	1.38×10^{-4}	3.03×10^{-1}
PreMRI_glcm_Idn	1.26×10^{-6}	1.98×10^{-5}
PreMRI_glcm_Idmn	5.96×10^{-6}	1.93×10^{-5}
PreMRI_glcm_Idm	1.68×10^{-3}	3.20×10^{-6}
PreMRI_glcm_Id	1.59×10^{-3}	3.67×10^{-6}
PreMRI_glcm_DifferenceVariance	2.96×10^{-4}	9.60×10^{-4}
PreMRI_glcm_DifferenceEntropy	1.94×10^{-3}	1.91×10^{-6}
PreMRI_glcm_DifferenceAverage	3.10×10^{-3}	1.74×10^{-5}
PreMRI_glcm_Correlation	2.15×10^{-3}	7.06×10^{-2}
PreMRI_glcm_Contrast	2.81×10^{-3}	1.82×10^{-3}
PreMRI_glcm_ClusterTendency	1.86×10^{-2}	3.04×10^{-2}
PreMRI_glcm_ClusterShade	2.47×10^{-3}	7.59×10^{-1}
PreMRI_glcm_ClusterProminence	1.72×10^{-2}	8.45×10^{-2}
PreMRI_glcm_Autocorrelation	4.26×10^{-8}	1.24×10^{-6}
PreMRI_firstorder_Variance	3.26×10^{-3}	1.14×10^{-2}
PreMRI_firstorder_Uniformity	4.22×10^{-2}	2.43×10^{-4}
PreMRI_firstorder_TotalEnergy	1.74×10^{-4}	1.95×10^{-1}
PreMRI_firstorder_Skewness	7.21×10^{-2}	6.18×10^{-1}
PreMRI_firstorder_RootMeanSquared	1.06×10^{-8}	3.49×10^{-8}
PreMRI_firstorder_RobustMeanAbsoluteDeviation	1.17×10^{-1}	1.96×10^{-3}
PreMRI_firstorder_Range	1.65×10^{-7}	2.69×10^{-6}
PreMRI_firstorder_Minimum	1.41×10^{-4}	4.69×10^{-3}
PreMRI_firstorder_Median	3.94×10^{-9}	3.06×10^{-8}
PreMRI_firstorder_MeanAbsoluteDeviation	1.31×10^{-2}	4.61×10^{-4}
PreMRI_firstorder_Mean	1.27×10^{-8}	4.47×10^{-8}
PreMRI_firstorder_Maximum	7.32×10^{-7}	3.60×10^{-7}
PreMRI_firstorder_Kurtosis	4.61×10^{-1}	1.27×10^{-1}
PreMRI_firstorder_InterquartileRange	2.04×10^{-1}	2.06×10^{-3}
PreMRI_firstorder_Entropy	7.18×10^{-3}	5.93×10^{-6}
PreMRI_firstorder_Energy	1.74×10^{-4}	1.95×10^{-1}
PreMRI_firstorder_90Percentile	6.73×10^{-8}	7.76×10^{-8}
PreMRI_firstorder_10Percentile	1.72×10^{-2}	1.10×10^{-5}
EarlyPE_glcm_SumSquares	1.76×10^{-1}	4.75×10^{-1}
EarlyPE_glcm_SumEntropy	3.88×10^{-2}	5.35×10^{-3}
EarlyPE_glcm_MaximumProbability	3.70×10^{-1}	6.37×10^{-1}
EarlyPE_glcm_JointEntropy	2.90×10^{-2}	1.92×10^{-2}
EarlyPE_glcm_JointEnergy	3.20×10^{-1}	1.47×10^{-1}
EarlyPE_glcm_JointAverage	3.15×10^{-7}	5.33×10^{-1}
EarlyPE_glcm_InverseVariance	1.19×10^{-3}	7.33×10^{-2}
EarlyPE_glcm_Imc2	2.44×10^{-6}	5.67×10^{-6}
EarlyPE_glcm_Imc1	1.00×10^{-8}	6.38×10^{-6}
EarlyPE_glcm_Idn	4.46×10^{-17}	5.60×10^{-4}
EarlyPE_glcm_Idmn	2.20×10^{-20}	5.56×10^{-5}

EarlyPE_glcm_Idm	1.21×10^{-3}	7.89×10^{-2}
EarlyPE_glcm_Id	1.01×10^{-3}	7.05×10^{-2}
EarlyPE_glcm_DifferenceVariance	3.15×10^{-1}	8.88×10^{-1}
EarlyPE_glcm_DifferenceEntropy	3.23×10^{-3}	1.16×10^{-1}
EarlyPE_glcm_DifferenceAverage	1.34×10^{-2}	1.25×10^{-1}
EarlyPE_glcm_Correlation	1.12×10^{-1}	4.20×10^{-4}
EarlyPE_glcm_Contrast	2.40×10^{-1}	6.80×10^{-1}
EarlyPE_glcm_ClusterTendency	1.26×10^{-1}	3.27×10^{-1}
EarlyPE_glcm_ClusterShade	5.55×10^{-1}	1.38×10^{-1}
EarlyPE_glcm_ClusterProminence	4.32×10^{-1}	4.18×10^{-1}
EarlyPE_glcm_Autocorrelation	1.88×10^{-6}	3.36×10^{-1}
EarlyPE_firstorder_Variance	1.23×10^{-1}	4.12×10^{-1}
EarlyPE_firstorder_Uniformity	1.02×10^{-1}	1.74×10^{-2}
EarlyPE_firstorder_TotalEnergy	2.08×10^{-4}	9.19×10^{-2}
EarlyPE_firstorder_Skewness	5.05×10^{-1}	3.87×10^{-1}
EarlyPE_firstorder_RootMeanSquared	3.23×10^{-8}	9.55×10^{-3}
EarlyPE_firstorder_RobustMeanAbsoluteDeviation	2.43×10^{-3}	7.43×10^{-3}
EarlyPE_firstorder_Range	3.17×10^{-3}	6.51×10^{-1}
EarlyPE_firstorder_Minimum	1.78×10^{-5}	4.03×10^{-1}
EarlyPE_firstorder_Median	1.96×10^{-9}	6.49×10^{-3}
EarlyPE_firstorder_MeanAbsoluteDeviation	4.76×10^{-3}	2.78×10^{-2}
EarlyPE_firstorder_Mean	9.98×10^{-9}	8.33×10^{-3}
EarlyPE_firstorder_Maximum	8.55×10^{-3}	6.88×10^{-2}
EarlyPE_firstorder_Kurtosis	7.32×10^{-2}	4.37×10^{-1}
EarlyPE_firstorder_InterquartileRange	4.60×10^{-3}	9.64×10^{-3}
EarlyPE_firstorder_Entropy	9.02×10^{-3}	1.67×10^{-2}
EarlyPE_firstorder_Energy	2.08×10^{-4}	9.19×10^{-2}
EarlyPE_firstorder_90Percentile	1.89×10^{-5}	9.39×10^{-3}
EarlyPE_firstorder_10Percentile	5.94×10^{-9}	1.75×10^{-2}
MiddleSER_glcm_SumSquares	4.29×10^{-2}	5.74×10^{-2}
MiddleSER_glcm_SumEntropy	8.61×10^{-4}	3.94×10^{-5}
MiddleSER_glcm_MaximumProbability	3.35×10^{-6}	8.54×10^{-4}
MiddleSER_glcm_JointEntropy	3.51×10^{-15}	3.15×10^{-5}
MiddleSER_glcm_JointEnergy	1.47×10^{-4}	3.06×10^{-3}
MiddleSER_glcm_JointAverage	6.40×10^{-4}	9.05×10^{-3}
MiddleSER_glcm_InverseVariance	1.25×10^{-3}	1.72×10^{-2}
MiddleSER_glcm_Imc2	7.98×10^{-18}	4.28×10^{-4}
MiddleSER_glcm_Imc1	7.11×10^{-30}	3.29×10^{-3}
MiddleSER_glcm_Idn	9.90×10^{-13}	1.15×10^{-4}
MiddleSER_glcm_Idmn	1.48×10^{-17}	2.73×10^{-3}
MiddleSER_glcm_Idm	1.06×10^{-3}	3.65×10^{-5}
MiddleSER_glcm_Id	5.64×10^{-4}	3.46×10^{-5}
MiddleSER_glcm_DifferenceVariance	3.70×10^{-1}	6.30×10^{-2}

MiddleSER_glcm_DifferenceEntropy	5.61×10^{-3}	2.72×10^{-5}
MiddleSER_glcm_DifferenceAverage	1.15×10^{-3}	3.89×10^{-5}
MiddleSER_glcm_Correlation	2.21×10^{-3}	5.93×10^{-3}
MiddleSER_glcm_Contrast	3.36×10^{-1}	5.67×10^{-2}
MiddleSER_glcm_ClusterTendency	1.42×10^{-2}	5.81×10^{-2}
MiddleSER_glcm_ClusterShade	3.67×10^{-1}	9.24×10^{-1}
MiddleSER_glcm_ClusterProminence	3.81×10^{-1}	3.07×10^{-1}
MiddleSER_glcm_Autocorrelation	3.57×10^{-2}	2.49×10^{-1}
MiddleSER_firstorder_Variance	2.11×10^{-2}	4.16×10^{-2}
MiddleSER_firstorder_Uniformity	1.41×10^{-3}	4.83×10^{-4}
MiddleSER_firstorder_TotalEnergy	1.13×10^{-3}	1.72×10^{-1}
MiddleSER_firstorder_Skewness	1.67×10^{-1}	6.85×10^{-1}
MiddleSER_firstorder_RootMeanSquared	7.83×10^{-8}	8.06×10^{-2}
MiddleSER_firstorder_RobustMeanAbsoluteDeviation	1.43×10^{-4}	5.90×10^{-5}
MiddleSER_firstorder_Range	5.09×10^{-2}	7.94×10^{-3}
MiddleSER_firstorder_Minimum	1.96×10^{-2}	9.10×10^{-3}
MiddleSER_firstorder_Median	4.42×10^{-7}	3.30×10^{-3}
MiddleSER_firstorder_MeanAbsoluteDeviation	1.64×10^{-4}	1.60×10^{-4}
MiddleSER_firstorder_Mean	7.86×10^{-8}	4.17×10^{-1}
MiddleSER_firstorder_Maximum	5.70×10^{-2}	1.42×10^{-2}
MiddleSER_firstorder_Kurtosis	1.33×10^{-1}	1.53×10^{-2}
MiddleSER_firstorder_InterquartileRange	2.61×10^{-4}	3.21×10^{-5}
MiddleSER_firstorder_Entropy	1.77×10^{-3}	6.37×10^{-5}
MiddleSER_firstorder_Energy	1.13×10^{-3}	1.72×10^{-1}
MiddleSER_firstorder_90Percentile	3.67×10^{-7}	4.61×10^{-4}
MiddleSER_firstorder_10Percentile	3.71×10^{-8}	2.96×10^{-4}
LateSER_glcm_SumSquares	5.91×10^{-3}	2.54×10^{-2}
LateSER_glcm_SumEntropy	7.29×10^{-5}	3.98×10^{-6}
LateSER_glcm_MaximumProbability	1.08×10^{-9}	1.85×10^{-3}
LateSER_glcm_JointEntropy	6.61×10^{-22}	7.08×10^{-6}
LateSER_glcm_JointEnergy	7.53×10^{-7}	3.00×10^{-3}
LateSER_glcm_JointAverage	2.32×10^{-4}	2.25×10^{-4}
LateSER_glcm_InverseVariance	2.20×10^{-3}	2.36×10^{-6}
LateSER_glcm_Imc2	8.53×10^{-14}	2.00×10^{-5}
LateSER_glcm_Imc1	2.38×10^{-31}	1.08×10^{-4}
LateSER_glcm_Idn	6.48×10^{-11}	5.21×10^{-5}
LateSER_glcm_Idmn	5.70×10^{-15}	1.44×10^{-3}
LateSER_glcm_Idm	2.51×10^{-3}	1.78×10^{-5}
LateSER_glcm_Id	1.47×10^{-3}	1.20×10^{-5}
LateSER_glcm_DifferenceVariance	1.46×10^{-1}	2.85×10^{-2}
LateSER_glcm_DifferenceEntropy	1.45×10^{-3}	2.05×10^{-6}
LateSER_glcm_DifferenceAverage	6.50×10^{-4}	2.47×10^{-6}
LateSER_glcm_Correlation	1.02×10^{-1}	6.43×10^{-2}

LateSER_glcm_Contrast	1.09×10^{-1}	2.71×10^{-2}
LateSER_glcm_ClusterTendency	6.87×10^{-4}	2.38×10^{-2}
LateSER_glcm_ClusterShade	2.54×10^{-1}	8.91×10^{-1}
LateSER_glcm_ClusterProminence	2.96×10^{-1}	3.13×10^{-1}
LateSER_glcm_Autocorrelation	2.84×10^{-2}	1.04×10^{-1}
LateSER_firstorder_Variance	2.51×10^{-2}	1.23×10^{-2}
LateSER_firstorder_Uniformity	1.24×10^{-3}	2.53×10^{-4}
LateSER_firstorder_TotalEnergy	1.12×10^{-3}	1.73×10^{-1}
LateSER_firstorder_Skewness	1.71×10^{-2}	9.42×10^{-1}
LateSER_firstorder_RootMeanSquared	8.30×10^{-11}	1.19×10^{-2}
LateSER_firstorder_RobustMeanAbsoluteDeviation	3.95×10^{-5}	2.77×10^{-5}
LateSER_firstorder_Range	4.29×10^{-3}	1.06×10^{-5}
LateSER_firstorder_Minimum	7.29×10^{-3}	2.27×10^{-4}
LateSER_firstorder_Median	5.83×10^{-10}	1.78×10^{-3}
LateSER_firstorder_MeanAbsoluteDeviation	1.62×10^{-5}	5.39×10^{-7}
LateSER_firstorder_Mean	8.49×10^{-11}	4.12×10^{-1}
LateSER_firstorder_Maximum	4.25×10^{-3}	1.85×10^{-5}
LateSER_firstorder_Kurtosis	2.56×10^{-2}	2.05×10^{-2}
LateSER_firstorder_InterquartileRange	5.63×10^{-5}	4.13×10^{-5}
LateSER_firstorder_Entropy	1.77×10^{-4}	6.15×10^{-6}
LateSER_firstorder_Energy	1.12×10^{-3}	1.73×10^{-1}
LateSER_firstorder_90Percentile	8.00×10^{-9}	1.29×10^{-4}
LateSER_firstorder_10Percentile	2.39×10^{-9}	1.07×10^{-5}

Note: PE for percentage enhancement, SER for signal enhancement ratio, and glcm for gray level co-occurrence matrix.

Table S2. Differentially enriched KEGG pathways between imaging subtypes 1 and 2 in the imaging-subtype discovery cohort (cutoff, FDR < 0.25).

**Description**	***P*-value**	**FDR**
Olfactory transduction	1.05×10^{-3}	4.64×10^{-2}
Cell cycle	1.25×10^{-3}	4.64×10^{-2}
Nucleocytoplasmic transport	1.27×10^{-3}	4.64×10^{-2}
Taste transduction	1.32×10^{-3}	4.64×10^{-2}
Drug metabolism - other enzymes	1.34×10^{-3}	4.64×10^{-2}
Chemical carcinogenesis - DNA adducts	1.36×10^{-3}	4.64×10^{-2}
Pentose and glucuronate interconversions	1.44×10^{-3}	4.64×10^{-2}
Porphyrin and chlorophyll metabolism	1.45×10^{-3}	4.64×10^{-2}
Ascorbate and aldarate metabolism	1.45×10^{-3}	4.64×10^{-2}
Biosynthesis of cofactors	2.46×10^{-3}	5.50×10^{-2}
Bile secretion	2.62×10^{-3}	5.50×10^{-2}
Metabolism of xenobiotics by cytochrome P450	2.67×10^{-3}	5.50×10^{-2}
Drug metabolism - cytochrome P450	2.71×10^{-3}	5.50×10^{-2}
Steroid hormone biosynthesis	2.75×10^{-3}	5.50×10^{-2}
Malaria	3.38×10^{-3}	5.50×10^{-2}
Intestinal immune network for IgA production	3.39×10^{-3}	5.50×10^{-2}
Basal cell carcinoma	3.72×10^{-3}	5.50×10^{-2}
Complement and coagulation cascades	4.10×10^{-3}	5.50×10^{-2}
Hematopoietic cell lineage	4.13×10^{-3}	5.50×10^{-2}
ECM-receptor interaction	4.20×10^{-3}	5.50×10^{-2}
Fanconi anemia pathway	4.26×10^{-3}	5.50×10^{-2}
Starch and sucrose metabolism	4.31×10^{-3}	5.50×10^{-2}
Viral protein interaction with cytokine and cytokine receptor	4.41×10^{-3}	5.50×10^{-2}
Ribosome biogenesis in eukaryotes	5.32×10^{-3}	6.27×10^{-2}
Retinol metabolism	5.46×10^{-3}	6.27×10^{-2}
Asthma	6.39×10^{-3}	7.05×10^{-2}
Salivary secretion	6.62×10^{-3}	7.05×10^{-2}
Focal adhesion	7.75×10^{-3}	7.96×10^{-2}
Hedgehog signaling pathway	1.06×10^{-2}	1.03×10^{-1}
MicroRNAs in cancer	1.08×10^{-2}	1.03×10^{-1}
Carbohydrate digestion and absorption	1.14×10^{-2}	1.05×10^{-1}
Cytokine-cytokine receptor interaction	1.25×10^{-2}	1.12×10^{-1}
Vascular smooth muscle contraction	1.49×10^{-2}	1.24×10^{-1}
Proteoglycans in cancer	1.54×10^{-2}	1.24×10^{-1}
Proteasome	1.56×10^{-2}	1.24×10^{-1}

Other glycan degradation	1.60×10^{-2}	1.24×10^{-1}
Dilated cardiomyopathy	1.65×10^{-2}	1.24×10^{-1}
PI3K-Akt signaling pathway	1.69×10^{-2}	1.24×10^{-1}
Ras signaling pathway	1.72×10^{-2}	1.24×10^{-1}
Amoebiasis	1.75×10^{-2}	1.24×10^{-1}
AGE-RAGE signaling pathway in diabetic complications	1.76×10^{-2}	1.24×10^{-1}
GABAergic synapse	1.85×10^{-2}	1.27×10^{-1}
Terpenoid backbone biosynthesis	2.16×10^{-2}	1.44×10^{-1}
Chemokine signaling pathway	2.21×10^{-2}	1.44×10^{-1}
Adrenergic signaling in cardiomyocytes	2.26×10^{-2}	1.44×10^{-1}
Osteoclast differentiation	2.45×10^{-2}	1.52×10^{-1}
Cell adhesion molecules	2.53×10^{-2}	1.52×10^{-1}
Biosynthesis of amino acids	2.53×10^{-2}	1.52×10^{-1}
Protein processing in endoplasmic reticulum	2.71×10^{-2}	1.54×10^{-1}
Pancreatic secretion	2.71×10^{-2}	1.54×10^{-1}
Calcium signaling pathway	2.75×10^{-2}	1.54×10^{-1}
Breast cancer	2.78×10^{-2}	1.54×10^{-1}
Hypertrophic cardiomyopathy	2.83×10^{-2}	1.54×10^{-1}
African trypanosomiasis	3.25×10^{-2}	1.73×10^{-1}
Rap1 signaling pathway	3.31×10^{-2}	1.73×10^{-1}
Homologous recombination	4.06×10^{-2}	2.08×10^{-1}

Table S3. Differentially enriched KEGG pathways between imaging subtypes 1 and 3 in the imaging-subtype discovery cohort (cutoff, FDR < 0.25).

**Description**	***P*-value**	**FDR**
Cell cycle	1.01×10^{-3}	1.65×10^{-1}
Salivary secretion	1.02×10^{-3}	1.65×10^{-1}
Taste transduction	2.04×10^{-3}	2.20×10^{-1}
Neuroactive ligand-receptor interaction	3.00×10^{-3}	2.42×10^{-1}

Table S4. Differentially enriched KEGG pathways between imaging subtypes 2 and 3 in the imaging-subtype discovery cohort (cutoff, FDR < 0.25).

**Description**	***P*-value**	**FDR**
Staphylococcus aureus infection	1.14×10^{-3}	1.11×10^{-1}
Neuroactive ligand-receptor interaction	2.04×10^{-3}	1.11×10^{-1}
Asthma	2.59×10^{-3}	1.11×10^{-1}
Terpenoid backbone biosynthesis	4.05×10^{-3}	1.11×10^{-1}
Ascorbate and aldarate metabolism	4.26×10^{-3}	1.11×10^{-1}
Pentose and glucuronate interconversions	4.42×10^{-3}	1.11×10^{-1}
Porphyrin and chlorophyll metabolism	4.78×10^{-3}	1.11×10^{-1}
Proteasome	5.03×10^{-3}	1.11×10^{-1}
N-Glycan biosynthesis	5.46×10^{-3}	1.11×10^{-1}
ECM-receptor interaction	5.72×10^{-3}	1.11×10^{-1}
Steroid hormone biosynthesis	5.81×10^{-3}	1.11×10^{-1}
Retinol metabolism	6.13×10^{-3}	1.11×10^{-1}
Drug metabolism - cytochrome P450	6.21×10^{-3}	1.11×10^{-1}
Chemical carcinogenesis - DNA adducts	6.25×10^{-3}	1.11×10^{-1}
Drug metabolism - other enzymes	6.54×10^{-3}	1.11×10^{-1}
Metabolism of xenobiotics by cytochrome P450	6.71×10^{-3}	1.11×10^{-1}
Other glycan degradation	6.99×10^{-3}	1.11×10^{-1}
Melanoma	7.03×10^{-3}	1.11×10^{-1}
Peroxisome	7.30×10^{-3}	1.11×10^{-1}
Bile secretion	7.69×10^{-3}	1.11×10^{-1}
Oxidative phosphorylation	7.81×10^{-3}	1.11×10^{-1}
Ribosome	9.90×10^{-3}	1.25×10^{-1}
Lysosome	9.90×10^{-3}	1.25×10^{-1}
ABC transporters	1.01×10^{-2}	1.25×10^{-1}
Biosynthesis of cofactors	1.15×10^{-2}	1.35×10^{-1}
Non-alcoholic fatty liver disease	1.18×10^{-2}	1.35×10^{-1}
Hepatitis C	1.30×10^{-2}	1.43×10^{-1}
Protein processing in endoplasmic reticulum	1.47×10^{-2}	1.56×10^{-1}
Diabetic cardiomyopathy	1.59×10^{-2}	1.63×10^{-1}
Protein digestion and absorption	1.71×10^{-2}	1.70×10^{-1}
Chemical carcinogenesis - reactive oxygen species	1.79×10^{-2}	1.72×10^{-1}
Thermogenesis	1.92×10^{-2}	1.75×10^{-1}
Lipid and atherosclerosis	1.96×10^{-2}	1.75×10^{-1}
Parkinson disease	2.00×10^{-2}	1.75×10^{-1}
Ubiquinone and other terpenoid-quinone biosynthesis	2.13×10^{-2}	1.80×10^{-1}
Prion disease	2.17×10^{-2}	1.80×10^{-1}

Carbohydrate digestion and absorption	2.59×10^{-2}	1.99×10^{-1}
Maturity onset diabetes of the young	2.73×10^{-2}	1.99×10^{-1}
Steroid biosynthesis	2.77×10^{-2}	1.99×10^{-1}
Ribosome biogenesis in eukaryotes	2.80×10^{-2}	1.99×10^{-1}
Breast cancer	2.83×10^{-2}	1.99×10^{-1}
Protein export	2.83×10^{-2}	1.99×10^{-1}
Necroptosis	2.91×10^{-2}	1.99×10^{-1}
Huntington disease	2.94×10^{-2}	1.99×10^{-1}

Supplementary Methods

RNA sequencing protocols

Tumor tissue were frozen and collected from 199 samples in the imaging-subtype discovery cohort. The total RNA was extracted by VAHTS Total RNA-seq (H/M/R) Library Prep Kit for Illumina in light of the manufacturer's protocol, and immediately frozen in liquid nitrogen and stored at -80℃. Ovation human FFPE RNA-seq library systems (NuGEN Technologies, San Carlos, CA, USA) was used to construct RNA-seq libraries, and RNA was sequenced on Illumina HiSeq X Ten platform (Illumina, San Diego, CA, USA) using paired-end 150 bp runs.
